# Long-Lived Termite Queens Exhibit High Cu/Zn-Superoxide Dismutase Activity

**DOI:** 10.1155/2018/5127251

**Published:** 2018-02-13

**Authors:** Eisuke Tasaki, Kazuya Kobayashi, Kenji Matsuura, Yoshihito Iuchi

**Affiliations:** ^1^Laboratory of Insect Ecology, Graduate School of Agriculture, Kyoto University, Kitashirakawa Oiwakecho, Kyoto 606-8502, Japan; ^2^Department of Applied Bioresources Chemistry, The United Graduate School of Agriculture, Tottori University, 4-101 Koyamacho-minami, Tottori 680-8553, Japan; ^3^Department of Biological Chemistry, Faculty of Agriculture, Yamaguchi University, 1677-1 Yoshida, Yamaguchi 753-8515, Japan; ^4^Hokkaido Forest Research Station, Field Science Education and Research Center, Kyoto University, 553 Tawa Shibecha-cho Kawakami-gun, Hokkaido 088-2339, Japan; ^5^Graduate School of Sciences and Technology for Innovation, Yamaguchi University, 1677-1 Yoshida, Yamaguchi 753-8515, Japan

## Abstract

In most organisms, superoxide dismutases (SODs) are among the most effective antioxidant enzymes that regulate the reactive oxygen species (ROS) generated by oxidative energy metabolism. ROS are considered main proximate causes of aging. However, it remains unclear if SOD activities are associated with organismal longevity. The queens of eusocial insects, such as termites, ants, and honeybees, exhibit extraordinary longevity in comparison with the nonreproductive castes, such as workers. Therefore, the queens are promising candidates to study the underlying mechanisms of aging. Here, we found that queens have higher Cu/Zn-SOD activity than nonreproductive individuals of the termite *Reticulitermes speratus*. We identified three Cu/Zn-SOD sequences and one Mn-SOD sequence by RNA sequencing in *R. speratus*. Although the queens showed higher Cu/Zn-SOD activity than the nonreproductive individuals, there were no differences in their expression levels of the Cu/Zn-SOD genes *RsSOD1* and *RsSOD3A*. Copper (Cu^2+^ and Cu^+^) is an essential cofactor for Cu/Zn-SOD enzyme activity, and the queens had higher concentrations of copper than the workers. These results suggest that the high Cu/Zn-SOD activity of termite queens is related to their high levels of the cofactor rather than gene expression. This study highlights that Cu/Zn-SOD activity contributes to extraordinary longevity in termites.

## 1. Introduction

Queens of termites, ants, and honeybees are representative eusocial insects that live up to 10 times longer than nonreproductive workers, although they possess the same genome [1–3]. Generally, longevity negatively correlates with reproduction among species [4]. Most animal species show a gradual decline in reproduction with age [5]. Indeed, sterile female flies have longer lifespans than fertile females [6], and germline ablation extends worm longevity [7]. However, the queens of eusocial insects are believed to live for long periods while also laying large numbers of eggs [8]. Because of their unique characteristics, eusocial insects are expected to facilitate the discovery of the mechanisms involved in longevity determination [9].

In most organisms, superoxide dismutases (SODs) are the first line of antioxidant defense against the toxic reactive oxygen species (ROS) that are generated by aerobic metabolism. SODs convert the toxic superoxide anion radicals into hydrogen peroxide, which is subsequently detoxified by catalase, glutathione peroxidase, and peroxiredoxin. At present, two types of Cu/Zn-SODs, which are localized to either cytoplasmic compartments (SOD1) or extracellular elements (SOD3), and Mn-SOD (SOD2), which is exclusively located in mitochondria, have been identified in a variety of organisms [10, 11]. The accumulation of ROS-mediated damage is associated with aging and negative effects on longevity [12, 13]. Therefore, SODs contribute to stress resistance associated with the lifespan of an organism. Indeed, many studies have reported that SODs contributed to the longevity of the fruit fly *Drosophila melanogaster* [14–16], the yeast *Saccharomyces cerevisiae* [17], and the nematode *Caenorhabditis elegans* [18]. However, there are also many conflicting reports [19–22]. The impact of high SOD activity levels on longevity remains to be determined.

Unlike short-lived organisms, such as fruit flies, yeasts, and nematodes, termites sustain their maturity for a long time and sexually reproduce each year, which leads to an extended period of strong selection that should promote long life for the reproductives (queens and kings). Therefore, termite reproductives may be suitable model organisms to investigate if SODs are proximate causes of longevity. *Reticulitermes speratus* is one of the most studied subterranean termites, with regard to its reproductive system [23], symbiotic system [24], pheromone communication [25, 26], and antioxidant system [27, 28]. In particular, a previous study revealed that *R. speratus* produced numerous neotenic queens by an asexual queen succession system [29], which results in adequate sample numbers for several experiments. Hence, we selected the termite *R. speratus* as a model organism.

In this study, we investigated if high expression of SODs is associated with the extreme lifespan of the queens of *R. speratus*. First, we identified three Cu/Zn-SOD sequences and one Mn-SOD sequence from the RNA sequencing (RNA-seq) data in *R. speratus*. Then, we tested if the levels of SODs are higher in termite queens than in nonreproductive individuals.

## 2. Materials and Methods

### 2.1. Termites

Animal ethics committee approval was not required for this study, which used insect species. Eight colonies of the termite *R. speratus* (workers, soldiers, nymphs, and mature neotenic queens) were collected from the experimental forest of Yamaguchi University, which is part of Mt. Himeyama in Yamaguchi, western Japan. We used pooled samples from different colonies for each experiment, as described in Table S1. These insect samples were preserved at −80°C until use.

### 2.2. Molecular Analysis of *R. speratus* SODs

The whole transcriptome of *R. speratus* was examined using next-generation RNA-seq technology in a previous study [26]. We obtained mRNA sequences of SOD genes from the transcriptome data by performing a BLAST search with the amino acid sequences of translated SOD genes in the termite *Zootermopsis nevadensis* and other model organisms (Table S2). The presence and location of signal peptide cleavage sites in SOD proteins were predicted using the SignalP 4.1 server [30] and TargetP 1.1 server [31, 32]. We performed multiple amino acid sequence alignments with CLUSTALW and conducted phylogenetic analyses using the molecular evolutionary genetics analysis software MEGA7 [33]. Gene evolutionary history was inferred using the maximum likelihood method based on the Whelan and Goldman model [34], which is the best model based on the Bayesian information criterion.

### 2.3. Protein Extraction

Whole insect body samples that had been stored at −80°C were ground to powder in liquid nitrogen, then homogenised by sonication in tubes with 20 mM Tris–HCl containing 2% protease inhibitor cocktail (*v*/*v*), and followed by centrifugation at 17,000 ×g for 10 min at 4°C. The supernatants containing proteins were transferred to new tubes and used as samples for analyses. For each sample, protein concentration was measured with a bicinchoninic acid assay kit before extraction. The protein samples were preserved at −80°C until use in antioxidant activity assays.

### 2.4. SOD Activity Assays

The activities of antioxidant enzymes were determined as in a previous report [35]. Briefly, we quantified SOD activity by using 2-(-4-iodophenyl)-3-(4-nitrophenyl)-5-(2,4-disulfophenyl)-2H-tetrazolium (WST-1; Dojindo) to detect superoxide anion radicals. The reaction mixture contained diluted xanthine oxidase (approximately 0.2 units), 0.1 mM xanthine, 25 *μ*M WST-1, 0.1 mM EDTA, and 50 mM Na_2_CO_3_ (pH 10.2) in a total volume of 3 mL. The absorbance at 438 nm was monitored at 30°C for 1 min. One unit was defined as the amount of enzyme required to inhibit 50% of an absorbance change of 0.06 per minute, which was equivalent to 0.8 units as determined by the standard procedure using cytochrome c. Mn-SOD activities were defined as 1 mM NaCN-resistant activity. We performed 6–9 biological replicates for queens and nonreproductive individuals of *R. speratus*.

### 2.5. Measurement of Copper Concentrations

Copper (Cu^2+^ and Cu^+^), which is a cofactor of Cu/Zn-SOD, was extracted from termite queens and workers with 0.1 M HCl and measured using Metallo Assay Copper LS (Metallogenics) according to the accompanying manual. The measurements were based on the DiBr-PAESA method. The concentrations of copper were calculated as the molecular weights of copper relative to the protein weights. Sixteen biological replicates were performed, each with a queen and four workers.

### 2.6. Quantitative Real-Time PCR

We designed primer pairs for each the SOD genes using Primer3 (version 1.1.4; [36]; Table S3). Using ISOGEN reagent (Nippon Gene), total RNA was extracted individually from the whole bodies of termite workers, soldiers, nymphs, and queens that had been frozen with liquid nitrogen and stored at −80°C until extraction. Immediately following extraction, cDNA was synthesized from the RNA using a PrimeScript™ RT reagent kit (Takara) and preserved at −20°C. Quantitative real-time PCR (qRT-PCR) was performed using a LightCycler® (Roche) with QuantiTect® SYBR® Green PCR reagents (Qiagen). All procedures were performed in accordance with each manufacturer's protocol. GAPDH was selected as the reference gene. Relative expression levels were calculated using a typical ∆∆Ct method. We performed 9–12 biological replicates for queens and nonreproductive individuals of *R. speratus*.

### 2.7. Statistical Analysis

R software package (version 3.2.2) was used for all statistical analyses. We performed unpaired *t*-tests followed by *P* value corrections using Holm's method for multiple comparisons. All data in graphs are presented as the mean ± standard error of the mean (SEM), and all calculated *P* values are provided in the figure legends. Differences were considered significant when the *P* value was <0.05.

## 3. Results

### 3.1. Identification of Cu/Zn-SOD and Mn-SOD Sequences in *R. speratus*

**Figure 1:**
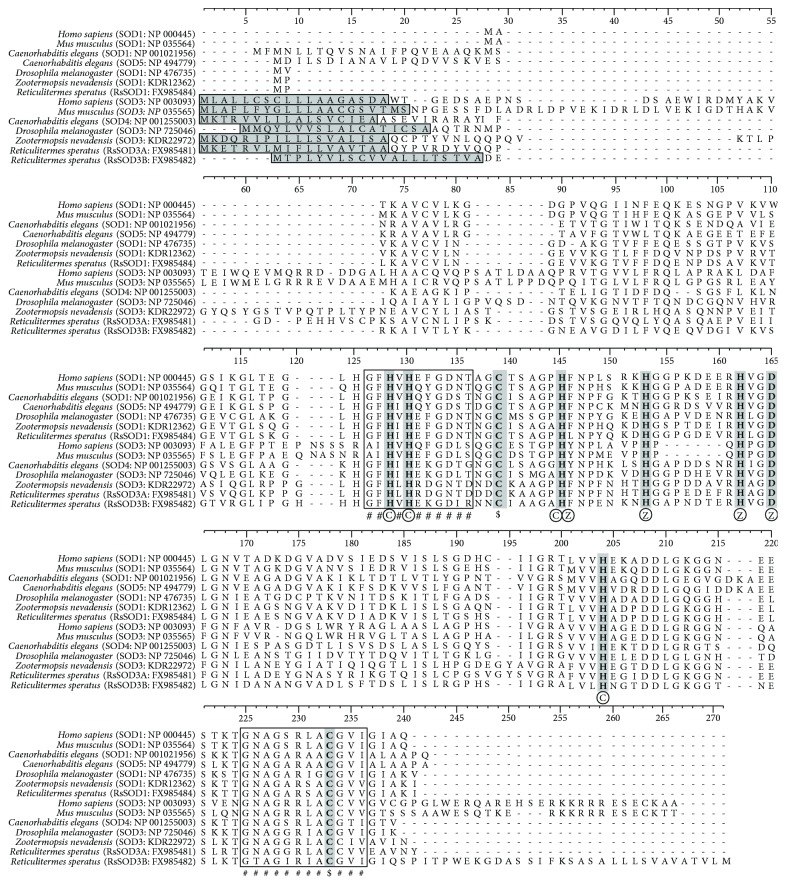
Alignment and structure conservation of Cu/Zn-SOD sequences. Multiple sequence alignment of *Homo sapiens*, *Mus musculus*, *Caenorhabditis elegans*, *Drosophila melanogaster*, *Zootermopsis nevadensis*, and *Reticulitermes speratus*. The signal cleavage site is indicated by a grey box. Conserved functional residues are highlighted in bold characters in grey (circled C = copper-binding site; circled Z = zinc-binding site; circled C, circled Z = copper- and zinc-binding sites; and $ = intrasubunit disulphide bridge site). SOD signature sequences are denoted in blocks with #.

We previously examined the whole transcriptome of *R. speratus* using next-generation RNA-seq technology [26]. Via a subsequent homology search of the known amino acid sequence data from the termite *Z. nevadensis* and several model insects, we inferred the existence of three Cu/Zn-SOD sequences and one Mn-SOD sequence in the *R. speratus* transcriptome (Table S2). We determined, by sequence alignments with the well-characterized SOD1 and SOD3 of *Homo sapiens*, *Mus musculus*, *C. elegans*, and *D. melanogaster*, that the *R. speratus* Cu/Zn-SODs had conserved all of the active sites that are essential for metal binding and disulphide formation [37], suggesting that they are active Cu/Zn-SODs (Figure 1). We also confirmed that the *R. speratus* Mn-SOD has a conserved manganese-binding site (Figure S1). Previous reports concluded that insects and other organisms have cytoplasmic and extracellular SODs [10, 38]. Our phylogenetic analysis revealed that one of the *R. speratus* sequences clustered with the cytoplasmic Cu/Zn-SODs of other organisms, whereas the other two sequences clustered with extracellular SODs (Figure S2). Furthermore, we determined that the predicted extracellular sequences have signal peptide cleavage sites (Figure 1 and Table 1). Although these phylogenetic analyses indicated that *R. speratus* has three Cu/Zn-SODs, we discarded *RsSOD3B* (FX985482) because of low reliability, based on the high E-value even in comparison to *Z. nevadensis* (Table S2). Thus, we treated *RsSOD1* (FX985484) as the cytoplasmic Cu/Zn-SOD gene, *RsSOD3A* (FX985481) as the extracellular Cu/Zn-SOD gene, and *RsSOD2* (FX985483) as the Mn-SOD gene in our analysis.

### 3.2. Termite Queens Have High Cu/Zn-SOD Activity in Comparison with Nonreproductive Individuals

**Figure 2:**
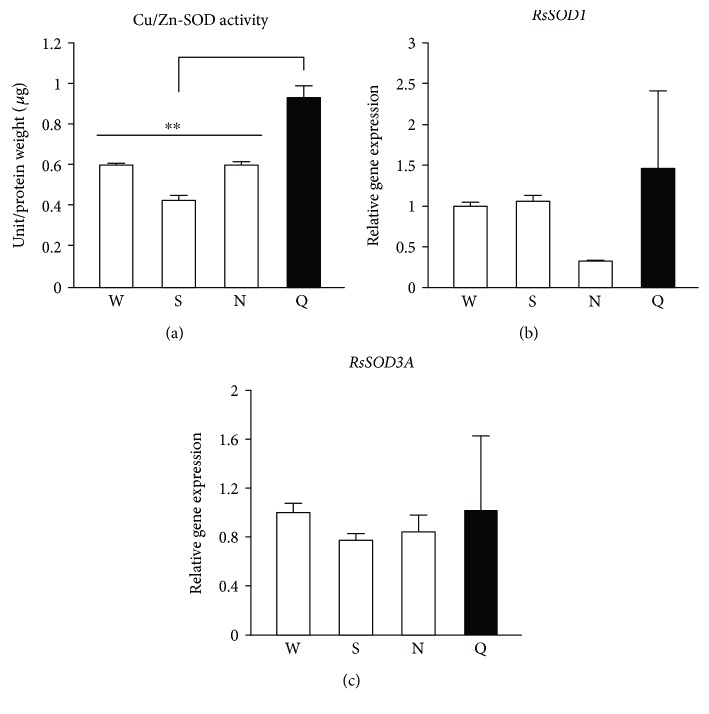
Cu/Zn-SOD activity and gene expression in *R. speratus* queens and nonreproductive individuals. (a) Queens (*n* = 9) showed higher Cu/Zn-SOD activity than nonreproductive workers (*n* = 6; *P* = 0.001), soldiers (*n* = 6; *P* < 0.001), and nymphs (*n* = 6; *P* = 0.001). (b) Queens (*n* = 9) did not have higher levels of *RsSOD1* gene expression than workers (*n* = 12; *P* = 1.000), soldiers (*n* = 12; *P* = 1.000), or nymphs (*n* = 12; *P* = 1.000). (c) Queens (*n* = 9) did not have higher levels of *RsSOD3A* gene expression than workers (*n* = 12; *P* = 1.000), soldiers (*n* = 12; *P* = 1.000), or nymphs (*n* = 12; *P* = 1.000). We used pooled samples, as described in Table S1, for several replications. White and black bars indicate nonreproductive individuals and queens, respectively. Error bars represent SEM. Significance was measured by unpaired *t*-test followed by Holm's adjustment (^∗∗^ *P* < 0.01). W: workers; S: soldiers; N: nymphs; Q: queens.

**Figure 3:**
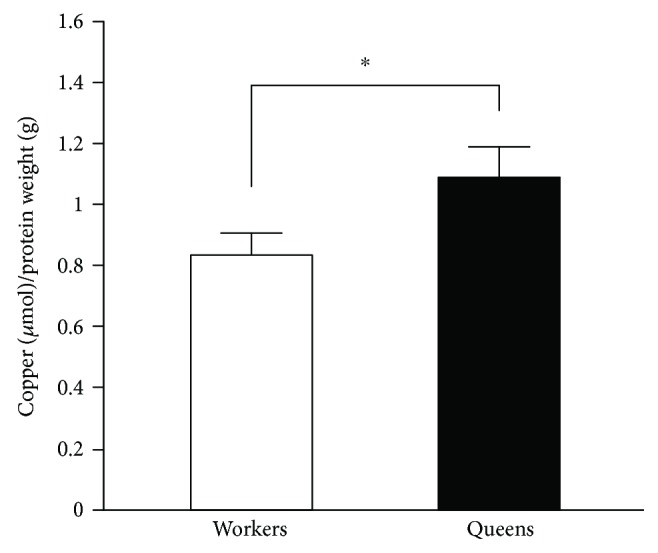
Measurement of copper concentrations in *R. speratus* queens and workers. Queens (*n* = 16) had significantly higher concentrations of copper than workers (*n* = 16; *P* = 0.038). We used pooled samples, as described in Table S1, for several replications. White and black bars indicate workers and queens, respectively. Error bars represent SEM. Significance was measured by unpaired *t*-test (^∗^ *P* < 0.05).

**Figure 4:**
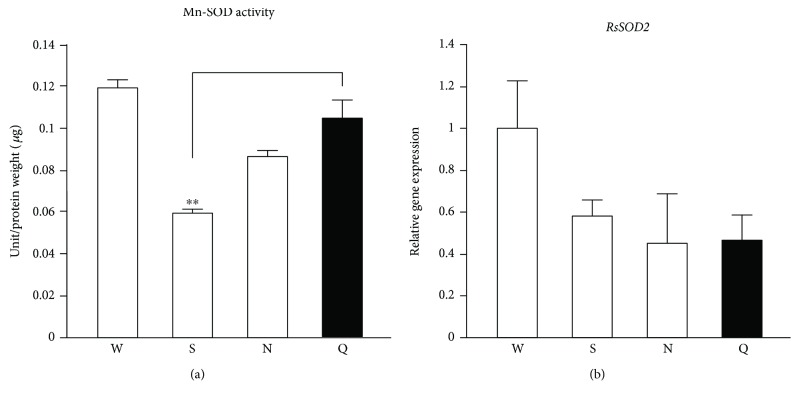
Mn-SOD activity and gene expression in *R. speratus* queens and nonreproductive individuals. (a) Queens (*n* = 9) had higher Mn-SOD activity than soldiers (*n* = 6; *P* = 0.004) but not workers (*n* = 6; *P* = 0.214) or nymphs (*n* = 6; *P* = 0.133). (b) Queens (*n* = 9) did not have higher levels of *RsSOD2* gene expression than workers (*n* = 12; *P* = 0.224), soldiers (*n* = 12; *P* = 0.813), or nymphs (*n* = 12; *P* = 0.960). We used pooled samples, as described in Table S1, for several replications. White and black bars indicate nonreproductive individuals and queens, respectively. Error bars represent SEM. Significance was measured by unpaired *t*-test followed by Holm's adjustment (^∗∗^ *P* < 0.01). W: workers; S: soldiers; N: nymphs; Q: queens.

Previously, we determined that *R. speratus* queens showed markedly lower levels of oxidative damage in comparison with nonreproductive workers, which is partly due to high catalase and peroxiredoxin expression and activity [27]. Therefore, we investigated if termite queens had higher SOD activities than nonreproductive individuals. We showed that Cu/Zn-SOD activity in *R. speratus* queens was higher than in nonreproductive individuals (Figure 2(a)). However, we found no differences in the expression of *RsSOD1* and *RsSOD3A*, which encode cytoplasmic Cu/Zn-SOD and extracellular Cu/Zn-SOD, respectively, between the queens and nonreproductive individuals (Figures 2(b) and 2(c)). We next examined if the copper concentrations corresponded to the Cu/Zn-SOD activity in termites. We found that termite queens have higher copper concentrations than nonreproductive workers (Figure 3). On the other hand, the Mn-SOD activity in *R. speratus* queens differed only from that in soldiers (Figure 4(a)). In addition, the queens and nonreproductive individuals had similar levels of *RsSOD2* expression (Figure 4(b)).

## 4. Discussion

To date, the determining factors of organismal longevity are among the most fascinating problems for many scientists. Eusocial insects have drawn attention as models for the study of the roles of specific genes in the aging process [9]. In the present study, we found that termite queens have higher Cu/Zn-SOD activity than nonreproductive individuals in the subterranean termite *R. speratus*. Given the long lifespan of termite queens, these findings suggest that high Cu/Zn-SOD activity may contribute to longevity. Our findings contract with those of earlier studies of eusocial Hymenoptera, which suggested that queen longevity was not associated with cytoplasmic Cu/Zn-SOD activity [39, 40]. In Hymenoptera, Cu/Zn-SOD is reportedly highly expressed in venom glands [41] and secreted in venom [38]. This unique trait may obscure a relationship between SOD levels and longevity. Although eusocial insects have attracted considerable attention for aging research, termites have not yet been investigated. This study highlights the importance of studying eusocial insects like termites, in addition to Hymenoptera, in the search for the mechanisms that contribute to the extraordinarily long lifespan of insect queens.

Oral fluid exchange (trophallaxis) is important for nutritional dynamics and communication in termites because queens depend on nestmate workers for most nutrients. Interestingly, we found that termite queens have higher copper concentrations than workers. Subterranean termites acquire several metal ions, including copper, from the soil and other foods [42]. This implies that higher copper concentrations in termite queens occur by trophic accumulation in castes. High copper concentrations are associated with increased Cu/Zn-SOD activity [43]. In the present study, we demonstrated that there were no significant differences in *RsSOD1* and *RsSOD3A* expression among the termite castes although the queens had higher Cu/Zn-SOD activity than nonreproductive individuals. There are often discrepancies between enzyme activity and gene expression levels. A previous report indicated that copper is critical for Cu/Zn-SOD activity and modulates enzymatic activity in the absence of changes in gene expression [43]. Therefore, the difference in copper concentrations between the queens and workers may be critical for their disparate levels of Cu/Zn-SOD activity. These results indicate that trophallaxis (from workers to queens) not only reduces the energy cost of foraging in termite queens but also provides nutritional benefits to the queens. Thus, trophallaxis may play an important role in nutrient accumulation contributing to termite queen longevity.

It remains unclear if increases in Cu/Zn-SOD activity are localized to specific tissues in termite queens. In mammals, ROS and SODs are thought to play important roles in several aspects of reproductive physiology because superoxide is generated and SOD is expressed in the ovary [44]. Additionally, several studies have reported a relationship between reproduction and antioxidant systems in insects [45–47]. Therefore, Cu/Zn-SOD may have an important role to play in reproductive processes, as well as in longevity, in termites. These findings suggest that Cu/Zn-SOD activity may be higher in reproductive tissues than in other somatic tissues. To determine if Cu/Zn-SOD is associated with reproduction in termites, future studies featuring tissue-specific analyses of Cu/Zn-SOD are required. Moreover, it is estimated that copper tends to accumulate in the reproductive tissues in termite queens. It is also necessary to determine the mechanisms by which copper transfer and accumulation occur in termite.

## Figures and Tables

**Table 1:** Predicted location and signal cleavage site of putative SOD proteins.

Gene sequence (database number)	Localization (reliability class)^a^	Signal cleavage
*Homo sapiens* (SOD1: NP 000445)	– (2)	None
*Mus musculus* (SOD1: NP 035564)	– (2)	None
*Caenorhabditis elegans* (SOD1: NP 001021956)	– (4)	None
*Caenorhabditis elegans* (SOD5: NP 494779)	– (2)	None
*Drosophila melanogaster* (SOD1: NP 476735)	– (2)	None
*Zootermopsis nevadensis* (SOD1: KDR12362)	– (2)	None
*Reticulitermes speratus* (RsSOD1: FX985484)	– (3)	None
*Homo sapiens* (SOD3: NP 003093)	Secreted (2)	1–18
*Mus musculus* (SOD3: NP 035565)	Secreted (2)	1–20
*Caenorhabditis elegans* (SOD4: NP 001255003)	Secreted (1)	1–17
*Drosophila melanogaster* (SOD3: NP 725046)	Secreted (1)	1–18
*Zootermopsis nevadensis* (SOD3: KDR22972)	Secreted (1)	1–18
*Reticulitermes speratus* (RsSOD3A: FX985481)	Secreted (1)	1–18
*Reticulitermes speratus* (RsSOD3B: FX985482)	Secreted (1)	1–20
*Homo sapiens* (SOD2: NP 001019636)	Mitochondrial (3)	1–24
*Mus musculus* (SOD2: NP 038699)	Mitochondrial (3)	1–24
*Caenorhabditis elegans* (SOD2: NP 492290)	Mitochondrial (4)	1–24
*Caenorhabditis elegans* (SOD3: NP 510764)	Mitochondrial (3)	1–24
*Drosophila melanogaster* (SOD2: NP 476925)	Mitochondrial (5)	1–17
*Zootermopsis nevadensis* (SOD2: KDR21306)	Mitochondrial (2)	1–80
*Reticulitermes speratus* (RsSOD2: FX985483)	Mitochondrial (3)	1–79

^a^TargetP classifies proteins as “secreted,” “mitochondrial,” or “other” (represented by “–” in the table). The reliability class (RC) ranges from 1 to 5, where a smaller number indicates a stronger prediction. RC is a measure of the difference (d) between the highest and the second highest output scores. There are 5 RCs, defined as follows: (1) d > 0.800, (2) 0.800 > d > 0.600, (3) 0.600 > d > 0.400, (4) 0.400 > d > 0.200, and (5) 0.200 > d.

## Supplementary Materials

Supplementary 1 Figure S1: alignment and structure conservation of Mn-SOD sequences. Multiple sequence alignment of *Homo sapiens*, *Mus musculus*, *Caenorhabditis elegans*, *Drosophila melanogaster*, *Zootermopsis nevadensis*, and *Reticulitermes speratus*. Conserved functional residues are highlighted in bold characters in grey (circled M = manganese-binding site).

Supplementary 2 Figure S2: maximum likelihood molecular phylogenetic tree of Cu/Zn-SOD sequences. We inferred the evolutionary history of the Cu/Zn-SODs using the maximum likelihood method based on the Whelan and Goldman model. The tree with the highest log likelihood (−2592.76) is shown. The percentage of trees in which the associated taxa clustered together is shown above the branches. Initial tree(s) for the heuristic search were obtained automatically by applying Neighbor-Joining and BioNJ algorithms to a matrix of pairwise distances estimated using a JTT model and then selecting the topology with a superior log likelihood value. A discrete gamma distribution was used to model the evolutionary rate differences among the sites (5 categories [+*G*, parameter = 1.8410]). The rate variation model allowed for some sites to be evolutionarily invariable ([+/], 21.40% sites). The tree is drawn to scale, with branch lengths measured in the number of substitutions per site. The analysis involved 14 amino acid sequences. All positions containing gaps and missing data were eliminated. There were a total of 137 positions in the final dataset. Evolutionary analyses were conducted in MEGA7.

Supplementary 3 Table S1: termite samples.

Supplementary 4 Table S2: target gene details.

Supplementary 5 Table S3: primer sequences.

## References

[1] Jemielity S., Chapuisat M., Parker J. D., Keller L. (2005). Long live the queen: studying aging in social insects. *Age*.

[2] Keller L., Genoud M. (1997). Extraordinary lifespans in ants: a test of evolutionary theories of ageing. *Nature*.

[3] Page R. E., Peng C. Y.-S. (2001). Aging and development in social insects with emphasis on the honey bee, *Apis mellifera* L. *Experimental Gerontology*.

[4] Partridge L., Gems D., Withers D. J. (2005). Sex and death: what is the connection?. *Cell*.

[5] Jones O. R., Scheuerlein A., Salguero-Gómez R. (2014). Diversity of ageing across the tree of life. *Nature*.

[6] Sgrò C. M., Partridge L. (1999). A delayed wave of death from reproduction in *Drosophila*. *Science*.

[7] Hsin H., Kenyon C. (1999). Signals from the reproductive system regulate the lifespan of *C. elegans*. *Nature*.

[8] Heinze J., Schrempf A. (2012). Terminal investment: individual reproduction of ant queens increases with age. *PLoS One*.

[9] Keller L., Jemielity S. (2006). Social insects as a model to study the molecular basis of ageing. *Experimental Gerontology*.

[10] Parker J. D., Parker K. M., Keller L. (2004). Molecular phylogenetic evidence for an extracellular Cu Zn superoxide dismutase gene in insects. *Insect Molecular Biology*.

[11] Zelko I. N., Mariani T. J., Folz R. J. (2002). Superoxide dismutase multigene family: a comparison of the CuZn-SOD (SOD1), Mn-SOD (SOD2), and EC-SOD (SOD3) gene structures, evolution, and expression. *Free Radical Biology & Medicine*.

[12] Dröge W. (2002). Free radicals in the physiological control of cell function. *Physiological Reviews*.

[13] Finkel T., Holbrook N. J. (2000). Oxidants, oxidative stress and the biology of ageing. *Nature*.

[14] Orr W., Sohal R. (1994). Extension of life-span by overexpression of superoxide dismutase and catalase in *Drosophila melanogaster*. *Science*.

[15] Parkes T. L., Elia A. J., Dickinson D., Hilliker A. J., Phillips J. P., Boulianne G. L. (1998). Extension of *Drosophila* lifespan by overexpression of human *SOD1* in motorneurons. *Nature Genetics*.

[16] Sun J., Tower J. (1999). FLP recombinase-mediated induction of Cu/Zn-superoxide dismutase transgene expression can extend the life span of adult *Drosophila melanogaster* flies. *Molecular and Cellular Biology*.

[17] Fabrizio P., Liou L. L., Moy V. N. (2003). *SOD2* functions downstream of Sch9 to extend longevity in yeast. *Genetics*.

[18] Melov S., Ravenscroft J., Malik S. (2000). Extension of life-span with superoxide dismutase/catalase mimetics. *Science*.

[19] Keaney M., Gems D. (2003). No increase in lifespan in *Caenorhabditis elegans* upon treatment with the superoxide dismutase mimetic EUK-8. *Free Radical Biology & Medicine*.

[20] Huang T. T., Carlson E. J., Gillespie A. M., Shi Y., Epstein C. J. (2000). Ubiquitous overexpression of CuZn superoxide dismutase does not extendlife span in mice. *The Journals of Gerontology: Series A, Biological Sciences and Medical Sciences*.

[21] Seto N. O., Hayashi S., Tener G. M. (1990). Overexpression of Cu-Zn superoxide dismutase in *Drosophila* does not affect life-span. *Proceedings of the National Academy of Sciences of the United States of America*.

[22] Orr W. C., Mockett R. J., Benes J. J., Sohal R. S. (2003). Effects of overexpression of copper-zinc and manganese superoxide dismutases, catalase, and thioredoxin reductase genes on longevity in *Drosophila melanogaster*. *The Journal of Biological Chemistry*.

[23] Matsuura K. (2017). Evolution of the asexual queen succession system and its underlying mechanisms in termites. *The Journal of Experimental Biology*.

[24] Ohkuma M. (2003). Termite symbiotic systems: efficient bio-recycling of lignocellulose. *Applied Microbiology and Biotechnology*.

[25] Matsuura K., Himuro C., Yokoi T., Yamamoto Y., Vargo E. L., Keller L. (2010). Identification of a pheromone regulating caste differentiation in termites. *Proceedings of the National Academy of Sciences of the United States of America*.

[26] Mitaka Y., Kobayashi K., Mikheyev A., Tin M. M. Y., Watanabe Y., Matsuura K. (2016). Caste-specific and sex-specific expression of chemoreceptor genes in a termite. *PLoS One*.

[27] Tasaki E., Kobayashi K., Matsuura K., Iuchi Y. (2017). An efficient antioxidant system in a long-lived termite queen. *PLoS One*.

[28] Tasaki E., Sakurai H., Nitao M., Matsuura K., Iuchi Y. (2017). Uric acid, an important antioxidant contributing to survival in termites. *PLoS One*.

[29] Matsuura K., Vargo E. L., Kawatsu K. (2009). Queen succession through asexual reproduction in termites. *Science*.

[30] Petersen T. N., Brunak S., von Heijne G., Nielsen H. (2011). SignalP 4.0: discriminating signal peptides from transmembrane regions. *Nature Methods*.

[31] Emanuelsson O., Nielsen H., Brunak S., von Heijne G. (2000). Predicting subcellular localization of proteins based on their N-terminal amino acid sequence. *Journal of Molecular Biology*.

[32] Nielsen H., Engelbrecht J., Brunak S., von Heijne G. (1997). Identification of prokaryotic and eukaryotic signal peptides and prediction of their cleavage sites. *Protein Engineering*.

[33] Tamura K., Dudley J., Nei M., Kumar S. (2007). MEGA4: molecular evolutionary genetics analysis (MEGA) software version 4.0. *Molecular Biology and Evolution*.

[34] Whelan S., Goldman N. (2001). A general empirical model of protein evolution derived from multiple protein families using a maximum-likelihood approach. *Molecular Biology and Evolution*.

[35] Iuchi Y., Okada F., Onuma K. (2007). Elevated oxidative stress in erythrocytes due to a SOD1 deficiency causes anaemia and triggers autoantibody production. *Biochemical Journal*.

[36] Rozen S., Skaletsky H. (2000). Primer3 on the WWW for general users and for biologist programmers. *Methods in Molecular Biology*.

[37] Perry J. J. P., Shin D. S., Getzoff E. D., Tainer J. A. (2010). The structural biochemistry of the superoxide dismutases. *Biochimica et Biophysica Acta (BBA) - Proteins and Proteomics*.

[38] Colinet D., Cazes D., Belghazi M., Gatti J. L., Poirié M. (2011). Extracellular superoxide dismutase in insects: characterization, function, and interspecific variation in parasitoid wasp venom. *The Journal of Biological Chemistry*.

[39] Parker J. D., Parker K. M., Sohal B. H., Sohal R. S., Keller L. (2004). Decreased expression of Cu-Zn superoxide dismutase 1 in ants with extreme lifespan. *Proceedings of the National Academy of Sciences of the United States of America*.

[40] Corona M., Hughes K. A., Weaver D. B., Robinson G. E. (2005). Gene expression patterns associated with queen honey bee longevity. *Mechanisms of Ageing and Development*.

[41] Peiren N., de Graaf D. C., Vanrobaeys F. (2008). Proteomic analysis of the honey bee worker venom gland focusing on the mechanisms of protection against tissue damage. *Toxicon*.

[42] Janzow M. P., Judd T. M. (2015). The termite *Reticulitermes flavipes* (Rhinotermitidae: Isoptera) can acquire micronutrients from soil. *Environmental Entomology*.

[43] Frendo J. L., Thérond P., Guibourdenche J., Bidart J. M., Vidaud M., Evain-Brion D. (2000). Modulation of copper/zinc superoxide dismutase expression and activity with in vitro differentiation of human villous cytotrophoblasts. *Placenta*.

[44] Sugino N. (2005). Reactive oxygen species in ovarian physiology. *Reproductive Medicine and Biology*.

[45] Michalkova V., Benoit J. B., Attardo G. M., Medlock J., Aksoy S. (2014). Amelioration of reproduction-associated oxidative stress in a viviparous insect is critical to prevent reproductive senescence. *PLoS One*.

[46] DeJong R. J., Miller L. M., Molina-Cruz A., Gupta L., Kumar S., Barillas-Mury C. (2007). Reactive oxygen species detoxification by catalase is a major determinant of fecundity in the mosquito *Anopheles gambiae*. *Proceedings of the National Academy of Sciences of the United States of America*.

[47] Diaz-Albiter H., Mitford R., Genta F. A., Sant’Anna M. R. V., Dillon R. J. (2011). Reactive oxygen species scavenging by catalase is important for female *Lutzomyia longipalpis* fecundity and mortality. *PLoS One*.

