# Supplementary material for: Long-Lived Termite Queens Exhibit High Cu/Zn-Superoxide Dismutase Activity
Source: Oxid Med Cell Longev. 2018 Feb 13;2018:5127251. doi: 10.1155/2018/5127251 (PMC5831368; doi:10.1155/2018/5127251)
Supplement: Supplementary 3 — Table S1: termite samples. [file 5127251.f3.docx]

**S1 Table. Termite samples.**

| Analysis | Colony ID | Termite samples  (pooled number of individuals per replications) |
| --- | --- | --- |
| SOD activities | YY140731A | Worker (10), soldier (8), nymph (5) and queen (4) |
| SOD activities | YY140914A | Queen (4) |
| SOD activities | YY141014A | Queen (4) |
| SOD gene expressions | YY130807A | Worker (3), soldier (3), nymph (2) and queen (1) |
| SOD gene expressions | YY151118A | Worker (3), soldier (3), nymph (2) and queen (1) |
| SOD gene expressions | YY140919A | Queen (1) |
| Copper measurement | KY160831A | Worker (4) and Queen (1) |
| Copper measurement | YY170605A | Worker (4) and Queen (1) |

Numbers in Colony IDs indicate the dates when the colonies were collected (e.g. colony YY140731A was collected on 31 July 2014).
