# Supplementary material for: Long-Lived Termite Queens Exhibit High Cu/Zn-Superoxide Dismutase Activity
Source: Oxid Med Cell Longev. 2018 Feb 13;2018:5127251. doi: 10.1155/2018/5127251 (PMC5831368; doi:10.1155/2018/5127251)
Supplement: Supplementary 4 — Table S2: target gene details. [file 5127251.f4.docx]

**S2 Table. Target gene details.**

| Target gene | Target gene ID | Accession no. | Query genes (Accession no.) | Query organisms | E-value | Identity |
| --- | --- | --- | --- | --- | --- | --- |
| *RsSOD1* | comp666385_c0_seq1 | FX985484 | Superoxide dismutase [Cu-Zn] (KDR12362) | *Z. nevadensis* | 1E–95 | 86% |
|  |  |  | Superoxide dismutase [Cu-Zn] (KDR22972) | *Z. nevadensis* | 2E–39 | 46% |
|  |  |  | Superoxide dismutase 1 (NP_001171498) | *A. mellifera* | 1E–78 | 75% |
|  |  |  | Superoxide dismutase 3 (ANS15098) | *A. mellifera* | 9E–49 | 57% |
|  |  |  | Superoxide dismutase [Cu-Zn] (NP_001037084) | *B. mori* | 2E–75 | 74% |
|  |  |  | Superoxide dismutase 1, isoform A (NP_476735) | *D. melanogaster* | 8E–74 | 70% |
|  |  |  | Superoxide dismutase [Cu-Zn]-like (NP_001156243) | *A. pisum* | 6E–76 | 72% |
|  |  |  | PREDICTED: superoxide dismutase [Cu-Zn] (XP_968284) | *T. castaneum* | 3E–82 | 80% |
| *RsSOD3A* | comp751766_c0_seq3 | FX985481 | Superoxide dismutase [Cu-Zn] (KDR22972) | *Z. nevadensis* | 1E−94 | 64% |
|  |  |  | Superoxide dismutase [Cu-Zn] (KDR12362) | *Z. nevadensis* | 2E−32 | 46% |
|  |  |  | Superoxide dismutase 1 (NP_001171498) | *A. mellifera* | 6E−34 | 47% |
|  |  |  | Superoxide dismutase 3 (ANS15098) | *A. mellifera* | 2E−34 | 44% |
|  |  |  | Superoxide dismutase [Cu-Zn] (NP_001037084) | *B. mori* | 6E−34 | 47% |
|  |  |  | Superoxide dismutase 3, isoform B (NP_610682) | *D. melanogaster* | 1E−29 | 41% |
|  |  |  | Superoxide dismutase 1, isoform A (NP_476735) | *D. melanogaster* | 4E−29 | 45% |
|  |  |  | Superoxide dismutase [Cu-Zn]-like precursor (NP_001156153) | *A. pisum* | 1E−34 | 48% |
|  |  |  | Superoxide dismutase [Cu-Zn]-like (NP_001156243) | *A. pisum* | 2E−32 | 45% |
|  |  |  | Superoxide dismutase [Cu-Zn]-like protein (KYB25410) | *T. castaneum* | 2E−32 | 44% |
|  |  |  | PREDICTED: superoxide dismutase [Cu-Zn] (XP_968284) | *T. castaneum* | 1E−31 | 46% |
| *RsSOD3B* | comp792253_c14_seq6 | FX985482 | Superoxide dismutase [Cu-Zn] (KDR12362) | *Z. nevadensis* | 9E−48 | 56% |
|  |  |  | Superoxide dismutase [Cu-Zn] (KDR22972) | *Z. nevadensis* | 2E−40 | 43% |
|  |  |  | Superoxide dismutase 1 (NP_001171498) | *A. mellifera* | 2E−49 | 57% |
|  |  |  | Superoxide dismutase 3 (ANS15098) | *A. mellifera* | 8E−41 | 53% |
|  |  |  | Superoxide dismutase [Cu-Zn] (NP_001037084) | *B. mori* | 2E−44 | 55% |
|  |  |  | Superoxide dismutase 3, isoform B (NP_610682) | *D. melanogaster* | 2E−42 | 50% |
|  |  |  | Superoxide dismutase 1, isoform A (NP_476735) | *D. melanogaster* | 4E−42 | 55% |
|  |  |  | Superoxide dismutase [Cu-Zn]-like precursor (NP_001156153) | *A. pisum* | 2E−65 | 53% |
|  |  |  | Superoxide dismutase [Cu-Zn]-like (NP_001156243) | *A. pisum* | 4E−46 | 54% |
|  |  |  | Superoxide dismutase [Cu-Zn]-like protein (KYB25410) | *T. castaneum* | 1E−49 | 54% |
|  |  |  | PREDICTED: superoxide dismutase [Cu-Zn] (XP_968284) | *T. castaneum* | 3E−47 | 56% |
| *RsSOD2* | comp776274_c1_seq2 | FX985483 | Superoxide dismutase [Mn], mitochondrial (KDR21306) | *Z. nevadensis* | 5E−155 | 90% |
|  |  |  | Superoxide dismutase 2, mitochondrial (NP_001171519) | *A. mellifera* | 1E−109 | 72% |
|  |  |  | Mn superoxide dismutase (NP_001037299) | *B. mori* | 2E−117 | 69% |
|  |  |  | Superoxide dismutase 2 (Mn), isoform A (NP_476925) | *D. melanogaster* | 2E−101 | 65% |
|  |  |  | PREDICTED: superoxide dismutase [Mn], mitochondrial (XP_972440) | *T. castaneum* | 7E−102 | 68% |

Blast search with the *R. speratus* sequences of translated SOD genes in *Zootermopsis nevadensis* (*Z. nevadensis*), *Apis mellifera* (*A. mellifera*), *Bombyx mori* (*B. mori*), *Drosophila melanogaster* (*D. melanogaster*), *Acyrthosiphon pisum* (*A. pisum*) and *Tribolium castaneum* (*T. castaneum*).
