# Supplementary material for: Long-Lived Termite Queens Exhibit High Cu/Zn-Superoxide Dismutase Activity
Source: Oxid Med Cell Longev. 2018 Feb 13;2018:5127251. doi: 10.1155/2018/5127251 (PMC5831368; doi:10.1155/2018/5127251)
Supplement: Supplementary 5 — Table S3: primer sequences. [file 5127251.f5.docx]

**S3 Table. Primer sequences.**

| Target gene | Sequence (5’–3’) | Amplicon (bp) |
| --- | --- | --- |
| *RsSOD1* | Forward; GGACCACATTTGAACCCTTACC | 122 |
|  | Reverse; TCAGCAATGTCAACTTTAGCAACTC |  |
| *RsSOD3A* | Forward; GGGCTACACGGTTTTCATCTTC | 146 |
|  | Reverse; GCCAATATGTTTCCAAAGTCTCCT |  |
| *RsSOD2* | Forward; CTACTGGCGGAAAACCAACC | 129 |
|  | Reverse; CAAGCCAACCCCAACCA |  |
| *RsGAPDH* | Forward; CCATAGAAAAGGCTTCTGCACATT | 89 |
|  | Reverse; AACAACAAACATTGGGGCATC |  |
